# Paroxysmal migraine associated with vancomycin administration: A case report, a review of pharmacovigilance databases for similar cases and possible mechanisms

**DOI:** 10.1097/MD.0000000000046545

**Published:** 2026-01-16

**Authors:** Jingjing Luo, Xinan Wu, Liuyi Ming, Liu Jun

**Affiliations:** a Department of Clinical Pharmacy, Hefei BOE Hospital, Hefei, China; b Department of Orthopaedics, Hefei BOE Hospital, Hefei, China.

**Keywords:** case report, drug-induced headache, episodic migraine, glycopeptide antibiotics, migraine, vancomycin

## Abstract

**Background:**

Vancomycin is a core drug for the treatment of methicillin-resistant *Staphylococcus aureus*, but its neurologic adverse effects are rarely reported. This case is the first to report a possible association between intravenous vancomycin and episodic migraine. Rapid identification of drug-induced migraine can avoid unnecessary invasive testing and medication delays and has direct clinical value.

**Objective:**

To report and document a case of episodic migraine that was highly correlated with the timing of intravenous vancomycin administration, assessing causality and its general clinical significance.

**Methods:**

This narrative case report is accompanied by an assessment of drug causality and supplemented by a review of pharmacovigilance databases. A systematic search using keywords was conducted. Inclusion criteria included case, cohort, or pharmacovigilance data related to headache/migraine use with glycopeptide drugs. Primary headaches unrelated to the drug were excluded.

**Case:**

A 54-year-old woman was admitted to the hospital with traumatic injuries resulting in bilateral lower extremity pain and a 1-hour bleeding episode on the left leg. The injuries included multiple fractures and traumatic injuries, and a postoperative wound infection developed.

**Results:**

Based on wound culture results, the patient was treated with intravenous vancomycin. During intravenous vancomycin treatment, the patient developed episodic migraines, and a causal relationship to vancomycin was confirmed using the Naranjo score. After discontinuation of vancomycin, the patient’s episodic migraines gradually resolved. The patient’s episodic migraines resolved completely within 3 days, and the same symptoms did not recur during subsequent treatment. The patient remained headache-free for 3 months after discharge.

**Conclusion:**

Pharmacovigilance data indicate that headaches associated with intravenous administration are rare but do occur with glycopeptide drugs. This review suggests that vancomycin may induce episodic migraines, a rare adverse effect that should be included in the differential diagnosis of acute headaches associated with intravenous glycopeptide drugs. Pharmacovigilance combined with therapeutic drug monitoring may serve as a low-cost, reproducible clinical identification tool. This case suggests a possible association between vancomycin and episodic migraines but does not directly prove causation, and further research is needed.

## 1. Introduction

### 1.1. Research background

Vancomycin is a glycopeptide antibiotic that remains the cornerstone of treatment for methicillin-resistant *Staphylococcus aureus* (MRSA) infections. Although documented adverse effects include nephrotoxicity, ototoxicity, and red man syndrome (incidence 3–15%),^[1]^ neurological complications are extremely rare. Episodic migraine (defined as sudden, severe headache accompanied by autonomic symptoms) has not been previously associated with vancomycin. This report describes the first case of vancomycin-induced migraine confirmed by a Naranjo causality assessment, supported by analysis of pharmacovigilance data from European databases.

### 1.2. Literature search and screening

A systematic search was conducted across PubMed, Embase, Web of Science, and CNKI from database creation to July 1, 2025. The search terms were a combination of subject terms and free words: (vancomycin OR glycopeptide OR teicoplanin) AND (headache OR migraine OR “drug-induced headache” OR “adverse reaction”).

No language restrictions were imposed. Inclusion criteria included: case reports, observational studies, or pharmacovigilance reports of headache/migraine associated with vancomycin/glycopeptides; reporting of route of administration, dose/concentration, or temporal association; and evidence of causality or de-/re-ignition.

Exclusion criteria included: primary headache or secondary headache unrelated to the drug; and animal or in vitro studies (nonclinical). Two researchers independently screened the citations and full texts; any disagreements were adjudicated by a third party. Extracted information included: population characteristics, dosing parameters, concomitant medications, temporal association, causality tool scores, outcomes, and follow-up.

### 1.3. Main experimental and medication conditions

The therapeutic drug monitoring (TDM) in this case was active monitoring, with trough concentration samples drawn 3 days after the first intravenous infusion to assess drug exposure and rule out concentration-related toxicity mechanisms. To facilitate reproducibility and clinical comparison, the key conditions of this case are listed here:

Dosing regimen: Vancomycin 1.0 g IV infusion every 8 hours; discontinued after headaches occurred, and switched to linezolid 0.6 g IV infusion every 12 hours.

TDM: Vancomycin trough concentration 18.2 μg/mL (usual target 10–20 μg/mL).

Concomitant medications: Morphine 5 mg IV was briefly used during the headache attack but provided limited relief.

De-challenge/re-challenge: Symptoms resolved after discontinuation of vancomycin but recurred after reinfusion.

Exclusionary studies: Head computed tomography, liver and kidney function, rheumatoid factor, and echocardiography were normal; magnetic resonance imaging/magnetic resonance venography on day 6 ruled out cerebral venous thrombosis.

Outcome and follow-up: Complete resolution 3 days after discontinuation of medication; no recurrence during hospitalization after switching antimicrobial regimen; and no recurrence 3 months after discharge.

## 2. Case presentation (history/examination)

A 54-year-old female patient was admitted to the hospital with trauma-induced bilateral lower extremity pain and 1-hour bleeding in the left leg. She was diagnosed with an open fracture of the tibia and fibula (left side), an intercondylar fracture of the femur (right side), a talus fracture (right side), metatarsal fractures (right third and fourth metatarsal bones), a cuneiform fracture (right side), and an avulsion of the thigh (right side). She had no history of allergies, asthma, atopic dermatitis, urticaria, or gastrointestinal disorders. She had no history of central nervous system disease, migraines, or chronic headaches. She denied any history of chronic anxiety, significant psychological stress, or sleep disorders.

## 3. Approaches (differential diagnosis, examination, and treatment)

### 3.1. Clinical timeline

Day 1: Emergency surgery for an open fracture.

Day 5: Postoperative wound infection developed, and wound secretions were cultured to identify MRSA. Vancomycin injection (1.0 g, IV, q8h) was administered for anti-infective treatment. Approximately 2 hours after stopping the vancomycin infusion, headache symptoms significantly resolved. Even after symptomatic treatment with opioid analgesics (prescribed morphine injection, 5 mg, intravenous push), headache symptoms remained unrelieved. The same symptoms recurred after another infusion of vancomycin.

**Figure 1.**
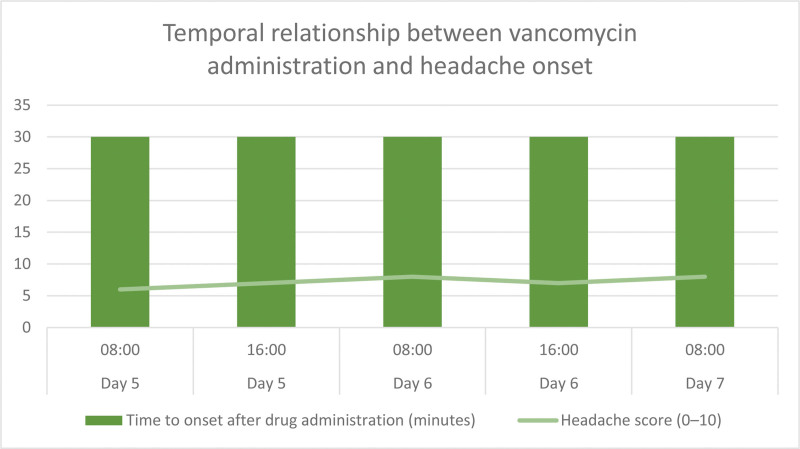
Temporal relationship between vancomycin administration and headache onset. Explanation: The graph shows the time correlation between vancomycin intravenous infusion and migraine onset during continuous treatment. The left side of the vertical axis represents the time (in minutes) of headache onset after drug infusion, and the right side represents the headache intensity score (0–10 points). This graph visually reflects the time dependence between drug administration and headache onset, supporting the possibility that vancomycin induces migraines.

Days 8–9: Due to the patient’s repeated complaints of unbearable headaches, second-line treatment was switched to linezolid (0.6 g, IV, q12h). The headache symptoms gradually resolved, and no further analgesics were used. Linezolid was chosen as an alternative medication based on the following criteria: it has considerable antimicrobial activity against MRSA; it has no known history of triggering migraine headaches; it is available in both oral and intravenous formulations, facilitating subsequent treatment transitions; and it is well tolerated by the patient. Day 10: The symptoms completely disappeared after switching to linezolid (see Figure 1 and Table 1 for details).

**Table 1:** Timeline and VAS score of vancomycin-related migraine cases.

Day	Event description	Medication status	VAS score
5	Postoperative infection was confirmed, and intravenous vancomycin 1.0 g q8h was started	Vancomycin	0
5.5	A unilateral throbbing headache occurred approximately 30 min after the first intravenous infusion	Vancomycin	8
6	Vancomycin treatment continued, and headaches recurred	Vancomycin	7
7	Stop vancomycin (pain relief is evident 2 h after stopping the drug)	Stopping medication	3
8	Recurrence of headache after re-administration of vancomycin	Vancomycin	7
8.5	Stop vancomycin and start linezolid 0.6g every 12 h	Linezolid	4
9	The headache continues to improve	Linezolid	2
10	The headache completely disappeared	Linezolid	0
3 days after discharge	Follow-up showed no recurrence of headache	No antibiotics	0
3 months after discharge	Follow-up showed no recurrence of headache	No antibiotics	0

### 3.2. Diagnostic examination

The following table lists the differential diagnosis and the symptoms that are present or absent in the patient (see Table 2 for details):

**Table 2:** Differential diagnosis and symptoms.

Diagnosis	Symptoms present	No symptoms
Medication overuse headache	No	Yes (Opioid use < 10 d)
Post-traumatic headache	No	Yes
Meningitis	No	Yes
Cerebral venous thrombosis	No	Yes

The patient did not undergo cerebrospinal fluid analysis. Physical examination revealed no signs of nuchal stiffness, Kernig’s sign, Brudzinski’s sign, or other meningeal irritation. Furthermore, based on the patient’s history and examination, common secondary headache causes, including pressure-related headaches, postoperative headaches, and intracranial hypotension headaches, were ruled out.

Head computed tomography, liver and kidney function, rheumatoid factor, and echocardiography were normal. Magnetic resonance imaging/magnetic resonance venography examination on day 6 ruled out cerebral venous thrombosis.

Vancomycin trough concentration: 18.2 μg/mL (target concentration: 10–20 μg/mL).

Differential diagnoses excluded: medication overuse headache (opioid use <10 days), post-traumatic headache, and meningitis.

The diagnosis of migraine in this case follows the International Classification of Headache Disorders, Third Edition criteria: recurrent, unilateral, throbbing headache lasting 4 to 72 hours; moderate or severe headache that interferes with daily activities; accompanied by at least one autonomic symptom, such as photophobia, phonophobia, or nausea or vomiting; and exclusion of alternative etiologies by imaging and laboratory tests. This patient met these criteria for the attack characteristics and exclusionary imaging findings (see Table 3 for details).

**Table 3:** Summary of diagnostic tests.

Inspection items	Result	Abnormal situation	Excluded diseases
Head CT	No abnormalities found	No	Intracranial mass or hemorrhage
MRI/MRV	No abnormalities found	No	Cerebral venous thrombosis
Liver and kidney function	Normal	No	Metabolic headache
Rheumatoid factor	Negative	No	Autoimmune diseases
Meningeal irritation signs	Negative	No	Meningitis, subarachnoid hemorrhage

CT = computed tomography, MRI = magnetic resonance imaging, MRV = magnetic resonance venography.

## 4. Results (outcomes and follow-up actions)

The patient’s headache symptoms resolved completely after 3 days and did not recur during subsequent treatment. The headache had not recurred during follow-up 3 months after discharge. The patient developed paroxysmal migraine during intravenous vancomycin treatment. The European Pharmacovigilance Database and the Naranjo Causality Assessment Tool confirmed that the patient, an adult female with no history of migraine, developed a severe, unilateral, throbbing headache within 30 minutes of the first intravenous infusion of vancomycin for a postoperative MRSA infection. The headache was not accompanied by autonomic symptoms such as photophobia or nausea. Symptoms resolved after discontinuation of the drug but recurred after reintroduction. The Naranjo score for this adverse reaction was 7 (indicating a “possible” causal relationship). The Naranjo score was used to confirm the causal relationship between this event and vancomycin use.

## 5. Discussion

In this case, we describe an association between onset time, double-positive rechallenge, and drug dose with the intensity of the reaction, suggesting a strong causal relationship between headache and vancomycin. Analysis using the Naranjo algorithm^[2]^ showed a “confirmed” relationship between vancomycin and throbbing headache.

Vancomycin-associated headache has not been previously reported in the literature. A review of suspected adverse drug reaction reports from the EudraVigilance (European Union Drug Safety Monitoring Database) European database from January 1, 2013, to September 16, 2023, reveals that headache accounted for 0.72% of all vancomycin adverse drug reaction (ADR) reports in EudraVigilance and 0.50% of all teicoplanin ADR reports. Female patients reported a higher incidence of headache with vancomycin than male patients, whereas male patients reported a higher incidence of headache with teicoplanin than female patients. Among routes of administration, intravenous administration had the highest incidence (see Table 4 for details).

**Table 4:** Headache caused by glycopeptides in EudraVigilance – European database of suspected adverse drug reactions (January 1, 2013 to September 16, 2023).

Medicine	Total number of reports (cases)	Number of headache reports (cases)	Headache as a proportion of ADR reports (%)	Proportion of female patients (%)	Proportion of male patients (%)	Proportion of headache after intravenous administration (%)
Vancomycin	13,900	100	0.72	Higher than men	–	Highest
Teicoplanin	8000	40	0.50	–	Higher than women	Highest

ADR = adverse drug reaction.

The temporal correlation and positive rechallenge/rechallenge pattern strongly suggest vancomycin-induced migraine. Potential mechanisms: Furthermore, vancomycin may induce pseudoallergic or anaphylactic reactions by directly stimulating mast cells.^[3]^Although histamine primarily mediates allergic symptoms, it may also trigger migraine, as has been reported in patients with systemic mastocytosis. Furthermore, opioid use may further enhance histamine release, exacerbating headache symptoms.^[4,5]^

Migraine aura is believed to be caused by cerebral vasoconstriction, unilateral gradual onset, and pulsatile (85%).^[6,7]^ Headache may be caused by activation of meningeal and vascular pain receptors, as well as changes in central pain regulation. According to a study by Sylvia S. Stefanos et al,^[8]^ vancomycin (pH 2.8–4.5) is acidic, and acidic drugs can cause vasoconstriction, which may be the cause of migraine. (Reference: Stefanos SS et al, 2023) Compared with oral vancomycin: There were no reports of migraine, which may be because first-pass metabolism reduces systemic exposure.

It should be noted that EudraVigilance data has underreporting bias; that is, milder or transient ADRs may not be reported, which may lead to an underestimate of the incidence. Therefore, our analysis results better reflect the proportion of cases that have been clinically identified and reported, rather than the true overall incidence.

### 5.1. Clinical application advantages

This study provides a “three-in-one” approach of time-locking, rechallenge/rechallenge, and TDM, which can significantly shorten the diagnosis time for drug-induced headache without increasing the cost of additional testing. This approach can be applied to the identification of ADRs associated with other intravenous antibiotics. For MRSA treatment, early identification and timely substitution of regimens (such as linezolid) can balance infection control and symptom relief, reducing the risk of overlapping analgesic medications.

### 5.2. Alternatives to existing therapies

This case does not attempt to replace existing standard therapy with “vancomycin discontinuation and drug switching.” Instead, it suggests that when suspected drug-induced migraine occurs, adjusting the infusion rate, pretreatment (such as antihistamines if necessary), or switching antimicrobials (such as linezolid) are evidence-based, reasonable, and replicable strategies.

With optimized dosing strategies (slow infusion, fractionated doses, small-volume pumping) combined with TDM, some patients can safely complete their treatment course. If symptoms become unbearable or recur, switching to a drug with the same antimicrobial spectrum is more reliable.

## 6. Conclusions

Vancomycin-associated migraine is rare (accounting for 0.72% in our analysis) yet warrants heightened clinical vigilance. We report a case of severe unilateral pulsatile migraine temporally associated with vancomycin administration. Given the increasingly widespread clinical use of vancomycin, prescribing clinicians should be alert to this potential ADR. Our findings suggest that this may reflect an effect shared by glycopeptide antibiotics.

To mitigate risk, we recommend the following measures: Pre-administration screening: Assess the patient’s history of headaches/migraines. Neurological monitoring: Closely observe for cranial or extracranial neurological symptoms during infusion; TDM: Measure serum trough concentrations to exclude toxicity-related mechanisms. These observations underscore the need for a systematic pharmacovigilance strategy when prescribing glycopeptide antibiotics.

In resource-limited healthcare settings, whether routine TDM monitoring should be implemented for all suspected vancomycin-associated headache cases warrants further discussion. From a cost-effective and feasible perspective, while TDM testing can help rule out adverse reactions due to elevated blood drug concentrations, its high cost and long testing cycle limit its clinical benefit for patients with mild or transient headaches. Therefore, in primary care settings or resource-constrained settings, initial identification of drug-related symptoms through clinical time-locking and drug discontinuation/re-challenge observation is a reasonable approach. TDM testing should only be considered to aid decision-making if symptoms persist, recur, or are accompanied by other systemic manifestations.

Overall, TDM has some clinical value in identifying vancomycin-associated migraine, but its implementation should be considered considering healthcare resource availability and patient specific circumstances, achieving a balance between scientific, cost-effective, and safety considerations.

This study has several limitations. First, this report is based solely on clinical observations from a single case. Although a strong causal association was established through temporal correlation, discontinuation-rechallenge testing, and the Naranjo score, these conclusions are insufficient to generalize to a wider population. Second, pharmacovigilance databases for adverse drug reactions (such as EudraVigilance) rely primarily on spontaneous reporting, which is subject to underreporting and reporting bias, and therefore the incidence rates reflected may underestimate the true incidence. Furthermore, this case cannot completely rule out the influence of other potential factors, such as postoperative stress, concomitant medications (such as opioid analgesics), or individual neurological susceptibility, which may all play a role in the development of headaches.

Thus, while this case provides preliminary evidence of a possible association between vancomycin and migraine, further validation is needed through multicenter case series or prospective drug monitoring studies to clarify the mechanism and clinical significance.

## Author contributions

**Conceptualization:** Jingjing Luo.

**Data curation:** Jingjing Luo.

**Formal analysis:** Jingjing Luo, Liuyi Ming.

**Funding acquisition:** Jingjing Luo, Liuyi Ming.

**Investigation:** Jingjing Luo, Xinan Wu.

**Methodology:** Jingjing Luo, Xinan Wu.

**Project administration:** Xinan Wu.

**Software:** Jingjing Luo.

**Validation:** Jingjing Luo.

**Visualization:** Jingjing Luo, Liu Jun.

**Writing – original draft:** Jingjing Luo.

**Writing – review & editing:** Jingjing Luo, Liuyi Ming.
